# Visualizing the Interpretation of a Criteria-Driven System That Automatically Evaluates the Quality of Health News: Exploratory Study of 2 Approaches

**DOI:** 10.2196/37751

**Published:** 2022-12-20

**Authors:** Xiaoyu Liu, Hiba Alsghaier, Ling Tong, Amna Ataullah, Susan McRoy

**Affiliations:** 1 Department of Computer Science University of Wisconsin Milwaukee Milwaukee, WI United States; 2 School of Health Sciences Southern Illinois University Carbondale Carbondale, IL United States; 3 Department of Health Informatics and Administration University of Wisconsin Milwaukee Milwaukee, WI United States

**Keywords:** health misinformation, machine learning, local interpretable model-agnostic explanation, LIME, interpretable artificial intelligence, AI

## Abstract

**Background:**

Machine learning techniques have been shown to be efficient in identifying health misinformation, but the results may not be trusted unless they can be justified in a way that is understandable.

**Objective:**

This study aimed to provide a new criteria-based system to assess and justify health news quality. Using a subset of an existing set of criteria, this study compared the feasibility of 2 alternative methods for adding interpretability. Both methods used classification and highlighting to visualize sentence-level evidence.

**Methods:**

A total of 3 out of 10 well-established criteria were chosen for experimentation, namely whether the health news discussed the costs of the intervention (the cost criterion), explained or quantified the harms of the intervention (the harm criterion), and identified the conflicts of interest (the conflict criterion). The first step of the experiment was to automate the evaluation of the 3 criteria by developing a sentence-level classifier. We tested Logistic Regression, Naive Bayes, Support Vector Machine, and Random Forest algorithms. Next, we compared the 2 visualization approaches. For the first approach, we calculated word feature weights, which explained how classification models distill keywords that contribute to the prediction; then, using the local interpretable model-agnostic explanation framework, we selected keywords associated with the classified criterion at the document level; and finally, the system selected and highlighted sentences with keywords. For the second approach, we extracted sentences that provided evidence to support the evaluation result from 100 health news articles; based on these results, we trained a typology classification model at the sentence level; and then, the system highlighted a positive sentence instance for the result justification. The number of sentences to highlight was determined by a preset threshold empirically determined using the average accuracy.

**Results:**

The automatic evaluation of health news on the cost, harm, and conflict criteria achieved average area under the curve scores of 0.88, 0.76, and 0.73, respectively, after 50 repetitions of 10-fold cross-validation. We found that both approaches could successfully visualize the interpretation of the system but that the performance of the 2 approaches varied by criterion and highlighting the accuracy decreased as the number of highlighted sentences increased. When the threshold accuracy was ≥75%, this resulted in a visualization with a variable length ranging from 1 to 6 sentences.

**Conclusions:**

We provided 2 approaches to interpret criteria-based health news evaluation models tested on 3 criteria. This method incorporated rule-based and statistical machine learning approaches. The results suggested that one might visually interpret an automatic criterion-based health news quality evaluation successfully using either approach; however, larger differences may arise when multiple quality-related criteria are considered. This study can increase public trust in computerized health information evaluation.

## Introduction

### Background

The internet has grown in popularity as a source for the public to learn about their health and investigate potential treatments for their health conditions. It is estimated that 80% of internet users consult web-based health information before making decisions [1]. Web-based media outlets such as social media feeds, forum threads, blogs, and newspapers have made information access and sharing easier. However, this has also accelerated the propagation of misleading information. Misinformation about health has been detected on different social media sites, such as Twitter [2-5], Facebook [6-9], YouTube [10-13], Pinterest [14,15], and Weibo [16,17]. Waszak et al [18] found that 40% of the most frequently shared links on social media contained medical information related to the most common diseases and causes of death were classified as fake news. In addition, the spread of health-related misinformation is not confined by geography. A series of studies have reported and studied health misinformation in different geographic settings, such as in the United States [19-21], China [16,17,22,23], India [24], and Italy [25,26]. With the rise of seeking health information on the internet, the concerns and health-related harm cases regarding misinformation have increased [27-29].

Unlike other types of misinformation, health-related misleading information, especially claims of efficacy about health interventions, such as medical treatments, tests, products, or procedures, can cause immediate actual harm to real people. The public and patients may be misled into making bad decisions that could result in severe consequences regarding people’s quality of life and even the risk of mortality. This negative influence has been observed in many countries worldwide, despite cultural, regulatory, and geographic variances [30]. When the COVID-19 pandemic started in 2019, health misinformation was further exacerbated globally as more people increasingly turned to social media to confirm possible symptoms and share treatment plans [31]. Misleading and erroneous information, information of low quality such as conspiracy theories, poorly sourced medical advice, and information trivializing the virus has not only contributed to widespread misconceptions about the novel coronavirus but also caused public panic, catastrophic consequences of public health, and even people’s distrust in public health institutions at the global level [32,33].

To address this public health crisis, continuing efforts to counteract health misinformation are being made across a wide range of disciplines and organizations. Detection and fact-checking work that relies on human effort is limited in scope, considering the high volume of fake news generated on the internet. Many attempts have been made to leverage artificial intelligence (AI) to analyze enormous amounts of information generated daily on a scale that would be impossible for humans to handle [34]. AI-powered automated detection methods, in comparison with people, are faster, more efficient, and may be deployed on targeted platforms at a low cost and on larger scale, by replicating human intelligence using data-driven analysis by computers [35]. When combating misinformation, AI technology may distinguish between accurate and misleading information using terms or word patterns associated with misinformation as cues from a relatively small set of articles that have been previously annotated by experts. Therefore, AI techniques can automate the process of detection of misleading information, which is conventionally performed manually.

### Related Work

In recent years, there has been an increasing trend in AI-based studies attempting to address health misinformation. The choice of health topic is a critical factor to consider, as it requires domain understanding and knowledge to assess the quality of health information and confirm the presence of misinformation. Health topics incorporated in past misinformation detection studies either focused on a specific topic, such as vaccination [36-38], Zika [39], autism [40], COVID-19 [41-44], or a collection of miscellaneous health conditions and lifestyle choices [45-50]. Health misinformation resides in various information outlets. Existing studies have proposed the detection of false, misleading health news on platforms such as Twitter [37,39,51,52], websites [36,45,46], and web-based forums [48,49].

Setting an appropriate benchmark for evaluating and annotating health information is unavoidable when developing detection systems. On the basis of the benchmark and objectives of this study, previous work on misinformation classification can be briefly categorized into a veracity-based approach or a criteria-based approach. Studies that follow a veracity-based approach involved training classifiers to assess the truth of each health-related claim using data that have been annotated to indicate whether the claim can be validated or refuted by finding a similar statement using a trusted source. These supporting sources might be experts from a third-party fact-checking organization (eg, Snopes [53]), medical and health-related professional organizations (eg, World Health Organization [54]), academic or research institutions (eg, John Hopkins Medicine [55]), and the federal government (eg, CDC [56]) which are typically considered as the officially sanctioned sources of bona fide accurate information and play an active role in myth debunking. For example, Ghenai and Mejova [39] proposed a novel pipeline that combines health experts, crowdsourcing, and machine learning (ML) to capture rumors on Twitter. The model was created using 13 million tweets concerning Zika infection between February 2016 and the Summer Olympics and rumors outlined by the World Health Organization and Snopes. The study found that rumor-related topics have a particularly burst behavior. The results demonstrated the feasibility of using automated techniques to remove rumor-bearing tweets when a questionable topic was detected.

In contrast, studies that followed the criteria-based approach looked at misinformation based on various quality-indicating criteria predefined by research. An example of such criteria might be the reliability or unreliability of the source; the rationale is that *intuitively, a news article published on an unreliable website and forwarded by unreliable users is more likely to be fake news than news posted by authoritative and credible users* [57]. For example, Liu et al [50] predefined a list of reliable and unreliable websites from which health-related articles from various sources on the Chinese Internet society were extracted for data set construction. Experiments were performed based on various ML classifiers using manually extracted features and text-classification modeling. The best performance among all models reached a precision of 0.8374. Other approaches were based on the idea that news that does not satisfy certain items on an assessment checklist for health information quality can be considered untrustworthy. For instance, Shah et al [37] used a 7-point checklist adapted from 2 validated tools, the DISCERN and Quality Index for health-related Media Reports checklists, to manually appraise the credibility of 474 web pages after sampling from 143,003 unique vaccine-related web pages shared on Twitter between January 2017 and March 2018. According to previous studies, the best-performing classifiers could distinguish between low, medium, and high credibility with an accuracy of 78% and labeled low-credibility web pages with a precision of >96%. Al-Jefri et al [58] and Afsana et al [59] both developed 10 classifiers to automatically evaluate the quality of health news based on the criteria developed by HealthNewsReview.org. However, the latter’s models demonstrated better classification performance owing to the inclusion of more features. In summary, veracity-based studies examined the authenticity of the news. The criteria-based approach focused on the characteristics of the news content, but the results did not make claims about the veracity of information.

In addition to the wide range of themes and strategies in detecting misinformation identified in the literature, methodologically, current studies also show the effectiveness of AI-based algorithms in classifying misinformation and quality information. Traditional ML algorithms, including Logistic Regression [40,47,52,60], support vector machine [37,40,47,50], decision tree [52,61], and random forest (RF) [37,39,41,48,60] have been widely applied in these studies, yielding effective and accurate performance. More recent studies have shown improved performance on large data sets by incorporating deep learning techniques, including convolutional neural networks [49,61], bidirectional encoder representations from transformers [43], and long short term memory [42,44,61]. As part of the modeling process, feature engineering has also been a critical step in improving the performance of classifiers. Zhao et al [57] reviewed and summarized 12 features used in health misinformation detection models. These features were grouped into 4 subsets: linguistic, topic, sentiment, and behavioral features.

Compared with traditional human fact-checking, an AI-based model consists of an algorithm that can automatically learn latent patterns and relationships from the data. However, one of the major challenges is the lack of a human-understandable rationale to support the results of classification tasks. Approaches that attempt to address this concern are often called “interpretable ML,” “explainable ML,” or “explainable AI” [62]. Open-source software with implementations of various interpretable ML methods are also available, such as local interpretable model-agnostic explanation (LIME) [63], Shapley Additive Explanations [64], Eli5 [65], and InterpretML [66], etc. These tools have been applied to various tasks, including image classification and text classification. With interpretations or visualized cues, users can verify the model and determine whether it meets their expectations. In addition, users can discover knowledge, justify predictions, and improve the performance of models using interpretable ML methods. Therefore, interpretable AI improves the trust and usability of the classifiers.

However, to date, only a small body of research has incorporated explainable AI models to combat health misinformation [43,67]. All of these studies on health information classification were veracity-based. A knowledge gap remains regarding the effectiveness of constructing an interpretable, criteria-driven classification system to help users evaluate the quality of health information.

### Objective of This Study

We aimed to address the aforementioned concerns and needs by creating an interpretable, criteria-driven system to assist the public in evaluating the quality of health news to mitigate the adverse consequences of health misinformation. Previous work using the HealthNewsReview.org data set and ML classifiers at the document level found that 3 criteria (cost, harm, and conflict) are more accurately classifiable among the 10 criteria, using linguistic features [58,68]; therefore, we selected these 3 criteria for this exploratory study. Our study, because it addressed interpretability, also focused on the use of features that are directly visualizable (linguistic features), excluding less visualizable features (such as average sentence length), which sometimes improved classification accuracy.

As an exploratory study, we opted to test 2 possible interpretation approaches, using 3 criteria. The evaluation results for the criteria will be visually explained with highlighted sentences as cues to enhance interpretability and reliability. As the number of highlighted sentences may affect the overall visual representation and effectiveness of the interpretation, we also attempted to determine the ideal range for the number of highlighted sentences.

## Methods

### Overview

**Figure 1:**
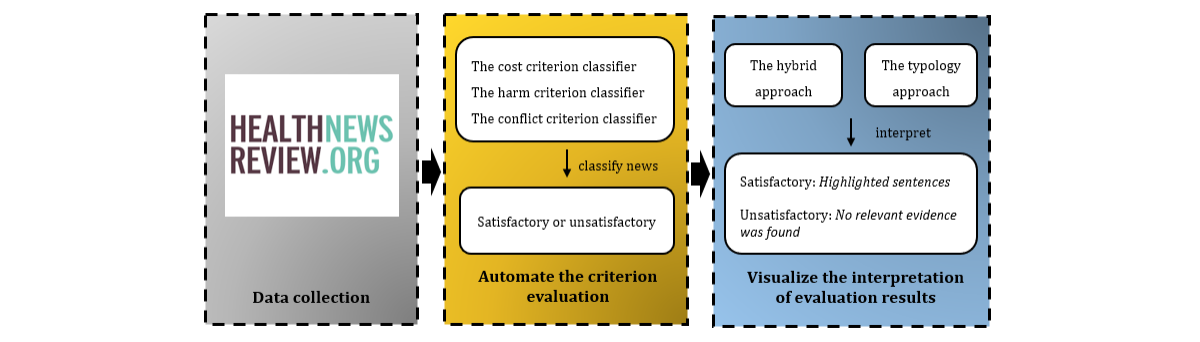
Overview of the exploratory experiment.

The experiment consisted of 3 components, as illustrated in Figure 1. In the first component, we collected reviewed health news from HealthNewsReview.org [69] to build the data set for modeling. Each criterion review result provided by HealthReview.org was treated as a classification target. The second component was a supervised document classification task that automated the criteria evaluation process. Each health news article was categorized automatically at the document level using established criteria and the output was binary (satisfactory or unsatisfactory). “Satisfactory” meant the entire health news met the given criterion and “unsatisfactory” meant the opposite.

The last component visualized and interpreted the evaluation results provided by the health news quality-evaluation system. For example, for the criterion “Does the news adequately explain or quantify the harms of the intervention?” the method highlighted sentences that described the harms of intervention to help users quickly understand how well the criterion was met.

We examined 2 approaches to achieve this goal. The first was a hybrid approach (the hybrid approach). It was inspired by principles from rule-based systems, where patterns are cospecified by LIME and experts. The second approach (the typology approach) was a supervised sentence typology classification method, where hand-labeled training data are analyzed algorithmically to build models that can detect similar patterns when applied to unseen data.

### Data Description and Collection

The data set that we used was adapted from an existing resource created by HealthNewsReview.org [69]. HealthNewsReview.org is a web-based project that reviewed articles from 2005 to 2018. Their team of experts rated the claims about health care interventions to improve the quality of health care information. Their rating instrument included 10 criteria used by the Australian and Canadian Media Doctor sites, and its interreviewer reliability was tested using a random sample of 30 stories [70]. HealthNewsReview.org included reviews of news stories from leading US media and news releases from institutes. The contents included efficacy claims about specific treatments, tests, products, or procedures. The news pieces were assessed using a standard rating system. At least 2 reviewers reviewed each news story. The reviewers were selected based on their years of experience in the health domain, spanning the fields of journalism, medicine, health services research, public health, or as patients, and each of them signed an industry-independent disclosure agreement. For each news story or news release reviewed, the criteria were scored as “satisfactory,” “unsatisfactory,” or “not applicable.” Total scores were posted for articles with ≤2 “not applicable” ratings and were expressed as proportions. It was acknowledged that increasing the diversity and independence of the reviewers could have reduced the potential for bias in the assessments. By the time the project ended, the website had accumulated 2616 health story reviews and 606 news release reviews.

For this study, we crawled health story news reviews and news release reviews, as archived by HealthNewsReviews.org, complying with the robots.txt. We scraped news contents that corresponded to the acquired reviews. Then, we visualized the results for the three selected criteria: (1) “Does the news adequately discuss the costs of the intervention?” (the cost criterion), (2) “Does the news adequately explain or quantify the harms of the intervention?” (the harm criterion), and (3) ***“***Does the news identify conflicts of interest?” (the conflict criterion).

### Automating the Criterion Evaluation

All 3 criteria applied to both news types, so we merged the 2 types of news content and treated them uniformly. We also combined health news that was scored as “unsatisfactory” or “not applicable” and named them as “unsatisfactory.” We preprocessed all news content via multiple text processing techniques, including removal of nonword elements (numbers, assented characters, and punctuation) and stop words, tokenization, stemming, and lemmatization. Then, we converted the textual representation into a vector space model using term frequency–inverse document frequency (TF-IDF).

We chose 4 representative algorithms: logistic regression, naive Bayes, support vector machine, and RF, from which we selected the best base algorithm that was suitable for automating the criterion evaluation. The 4 algorithms are commonly used in health misinformation classification tasks, as evident in previous studies [36,38,39,46,51,59], and were found to be effective. We applied RandomSearch to determine the optimal model hyperparameters for building the classifier. For our study, we defined the best classifier output from RandomSearch as the feature count, hyperparameter, and algorithm combination that produced the highest mean 5–cross-validated area under the curve (AUC) score. The performance of the classifier was further evaluated through 50-repeated 10–fold-validation.

### Visualizing the Interpretation of Evaluation Result

We experimented with 2 approaches to visualize the interpretation of the evaluation results. The desired outcome was that all highlighted sentences were relevant to the examined criterion and provided evidence to assist end users in comprehending and validating the evaluation results. To determine what qualified a sentence as evidence, we strictly adhered to the criteria definitions and review guidelines provided by HealthNewsReview.org [71-73]. For example, as per the explanation of the harm criterion provided by HealthNewsReview.org, satisfactory health news on the harm criterion should “include a discussion of harms and side effects, as well any measured ‘adverse events’ in a study” [71]. The measured “adverse events” can be addressed by a discussion of “both frequency of side effects and severity of side effects” and a discussion of “both major and minor side effects” [71].

#### The Hybrid Approach

The hybrid approach combined the interpretable AI technique, LIME, rule-based systems, and supervised document classification. LIME, proposed in 2016 by Ribeiro et al [74], belonged to a family of local model-agnostic methods, a type of interpretable AI method. It is used to explain the individual predictions of black-box ML based on a surrogate model, which is trained to approximate the predictions of the underlying black-box model [74,75]. The intuition of LIME is based on the idea that the behavior of a black-box model can be learned by perturbing the input. Specifically, a modified data set is generated by LIME through permutation by removing word features, corresponding to which predictions are obtained from the black-box model. Words with feature weights >0 indicate that the removal of such words affects the prediction result. For a negative case, no nonzero weight was estimated because regardless of which word was removed, the predicted evaluation result remained the same. Thus, an explanation can be generated by approximating the underlying model with a more interpretable model (such as a linear model or decision tree), learned locally on perturbations of the original instance [75]. Owing to the local fidelity nature of LIME, it does not guarantee a good global approximation [76]. A critique LIME often receives is that it lacks “stability” [77]. There are cases in which the surrogate model built by LIME can predict the instance correctly but provide incorrect reasons [75]. To address the instability of LIME, adding manually selected keywords can reduce the risk of obtaining incorrect keywords for highlighting. In this approach, we adopted the LIME method to facilitate the interpretable result of the predicted criterion evaluation. The Python packages used for implementing LIME algorithms were ELI5 [65] and LIME [63] application programming interface packages.

The explanation of the classification model for each criterion using the hybrid approach consisted of 3 steps. First, an ML classifier classifies health news as satisfactory or unsatisfactory based on the chosen criterion. Then, the classification model learned the difference of word distribution in satisfactory or unsatisfactory instances from the collection of health news document sets. LIME highlighted keywords in texts that contributed to the prediction. The keywords were also ranked using a weighted score, indicating their contribution to the prediction. Finally, we combined the keywords that contributed to a satisfactory prediction with a list of manually selected keywords, as shown in Table 1. The manual selection of the keywords was based on a consensus among the annotators who had taken part in the processes of evidence extraction for the typology approach.

We then extended the highlighting from the keyword to the sentence level to enhance the final visual representation. Sentences containing keywords with more weight were prioritized for highlighting. By default, manually selected keywords outweighed any keywords automatically picked by LIME.

**Table 1:** Lists of manually selected keywords for the cost, harm, and conflict criteria.

Criterion	Manually selected keywords
The cost criterion	Price, cost, charge, insurance, and pay
The harm criterion	Side effect, adverse reaction, adverse event, complication, and risk
The conflict criterion	Fund, sponsor, grant, spokesman, professor, and director

#### The Typology Approach

The typology approach was a sentence-level text-classification task. This approach was inspired by the study of persuasive communication and rhetoric. Reynolds and Reynolds [78] distinguished between statistical, testimonial, anecdotal, and analogical evidence. Hoeken and Hustinx [79] put forward 4 types of evidence in argumentation: individual examples, statistics, causal explanations, and expert opinions. Subsequent studies showed that machines can detect various types of evidence. For example, Fiok et al [80] built a classification model to automatically identify the evidence of respect in Twitter communication. There were 2 types of sentences in each health news item in our study. In the harm criterion, the first type of sentences was the evidence that supported the predicted evaluation result. Sentences of this type contained a description of side effects, including the symptoms, severity, and frequency of the symptoms. The second type of sentence referred to those that could not justify why a piece of certain health news satisfied a given criterion. Therefore, they were not characterized as evidence.

To implement the typology approach, for each criterion task, we designed and experimented with the typology approach in 2 stages. The first stage was to build an annotated data set of the sentence evidence. We extracted sentence evidence from health news that was evaluated as satisfactory by HealthNewsReview.org. A total of 3 people performed the sentence extraction tasks. The project investigator provided training and clarification to the other 2 extractors. The sentence extraction guideline fully adopted the criteria explained by HealthNewsReivew.org [71-73]. Two people performed most of the extraction work. Another individual worked as an independent reviewer to resolve disagreement. When combining the extracted sentences, sentences picked by the 2 extractors were characterized as evidence. If a sentence was extracted by an extractor but not picked by the other, an independent third person was invited to resolve the disagreement. All approved sentences were considered positive. To build the negative class, we randomly selected the same number of sentences irrelevant to the evaluation of the pertinent criterion. An interannotator agreement was assessed using both simple counts and the percentage of the final quantity of the evidence in the total extracted items to address the relatively small sample size. The interrater agreement was also in line with the expectations of other studies [81]. The second stage involved building a supervised ML classifier. We followed the same steps as for automating the criterion evaluation.

For the final visual representation, the sentence classifier was applied to health news content to identify sentence evidence. Sentences with a higher probability of being categorized as evidence by the classifier were prioritized for highlighting purposes.

#### Evaluating and Optimizing the 2 Approaches

For each criterion’s interpretation, we evaluated 2 visualization approaches to determine how accurately each scheme highlighted the sentences that supported the prediction result. The evaluation was conducted using 20 test cases. The selection of 20 test cases was based on the observation that the true positive health news counts in the test set (30% of the data set) ranged from 20 to 70, depending on the task criterion type. We measured the accuracy of 2 highlighting schemes by calculating the percentage of correctly highlighted evidence for all highlighted sentences. A total of 3 people evaluated the correctness of the highlighted sentence in accordance with each criterion’s guideline. An independent reviewer was invited to handle any disputes.

As the number of highlighted sentences may affect the highlighting accuracy and thus the final visual representation, we calculated a spectrum of accuracies of both the highlighting approaches when the number of highlighted sentences increased from 1. A threshold was then selected with the lowest accuracy to determine the optimal range of sentence counts for highlighting.

## Results

### Classification Model Performance

After removing dead links (to inaccessible news content), the acquired data set yielded 1453 stories and 579 news releases. Among the 2032 health news instances, the satisfactory or unsatisfactory instance ratios for the cost, harm, and conflict criteria were 25.03% (405/1618), 44.71% (625/1398), and 98.14% (1002/1021), respectively. Of the 4 experimental algorithms, RF was found to be the most effective in automating the evaluation of all the 3 criteria, as shown in Multimedia Appendix 1, despite the fact that the feature count varied according to the criterion. Table 2 shows the set of optimal hypermeters that RandomSearch selected for each criterion classifier.

**Figure 2:**
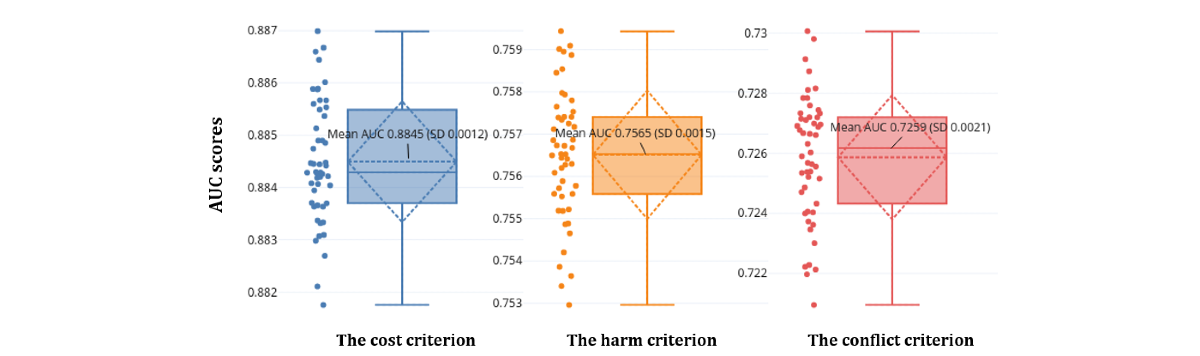
The performance of the cost, harm, and conflict criterion classifiers was measured with 10-fold cross-validated area under the curve (AUC) scores with a total of 50 repetitions.

For the cost, harm, and conflict criteria, Figure 2 shows that the average AUCs were 0.8845, 0.7565, and 0.7259, respectively, after 50 repeated 10-fold validations.

**Table 2:** Hyperparameters selected by RandomSearch for each criterion evaluation classifier.

Criteria	Base classifier	Word feature count, n	Hyperparameters
The cost criterion	Random forest	1000	(“n_estimators”: 600, “min_samples_split”: 2, “min_samples_leaf”: 4, “max_features”: “sqrt,” “max_depth”: 10, and “bootstrap”: false)
The harm criterion	Random forest	2000	(“n_estimators”: 1400, “min_samples_split”: 10, “min_samples_leaf”: 4, “max_features”: “auto,” “max_depth”: 90, “bootstrap”: false)
The conflict criterion	Random forest	1000	(“n_estimators”: 1200, “min_samples_split”: 10, “min_samples_leaf”: 1, “max_features”: “auto,” “max_depth”: 20, and “bootstrap”: true)

### Interpretable Model Performance

#### The Visual Interpretation by the Hybrid Approach

**Figure 3:**
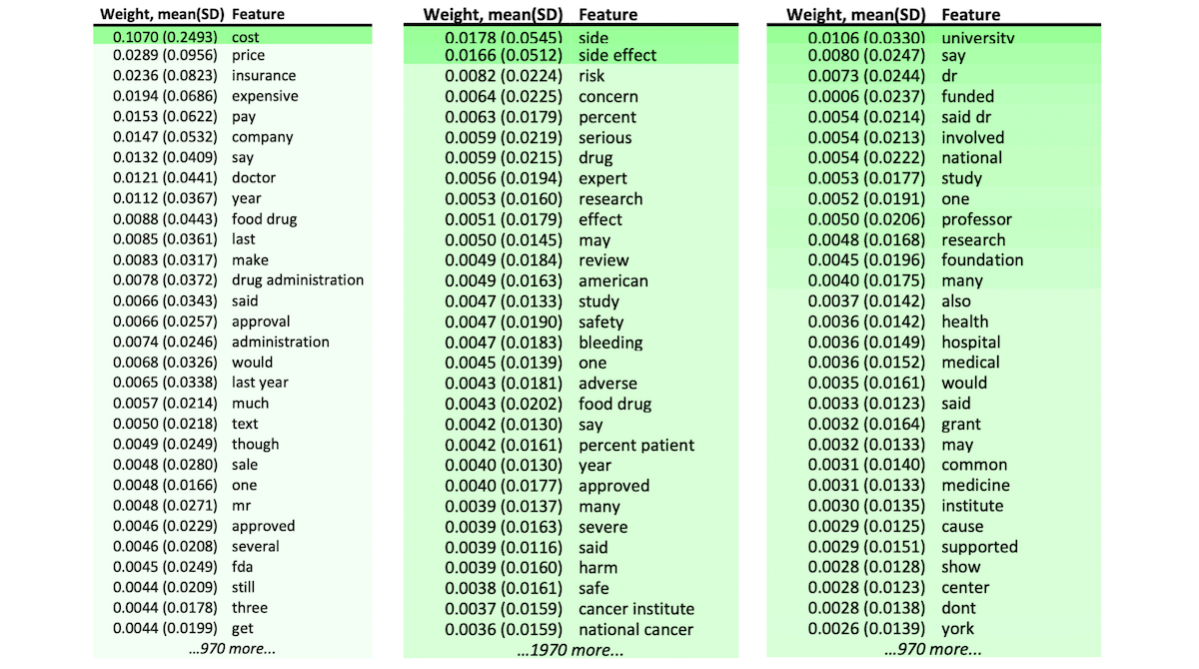
Top 30 word features with their feature weights in 3 criteria (the cost, harm, and conflict criteria) classifiers. The word feature weights signify how much discriminatory information each word contributes to the classification task by random forest algorithm.

The LIME Text Explainer visualized how different word features contributed to the evaluation results for each classifier. Figure 3 illustrates the top 30 bigram or unigram word features that contributed to the classification learned from the entire data set related to a given criterion. For example, the binary word feature with the highest weight in the harm criterion classification was “side effect.” Words that directly indicate the harm of intervention, such as “risk,” “concern,” “bleeding,” and “harm,” also ranked among the top features. Similarly, words that are commonly used to describe the intervention costs and insurance coverage such as “cost,” “insurance,” “expensive,” and “pay” were also observed high in contribution to the evaluation for the cost criterion. For the conflicts criterion, the words were descriptive of one’s affiliations such as “university,” “dr,” and “professor” stand out. The keyword “funded,” which directly discloses funding information, also ranked high.

**Figure 4:**
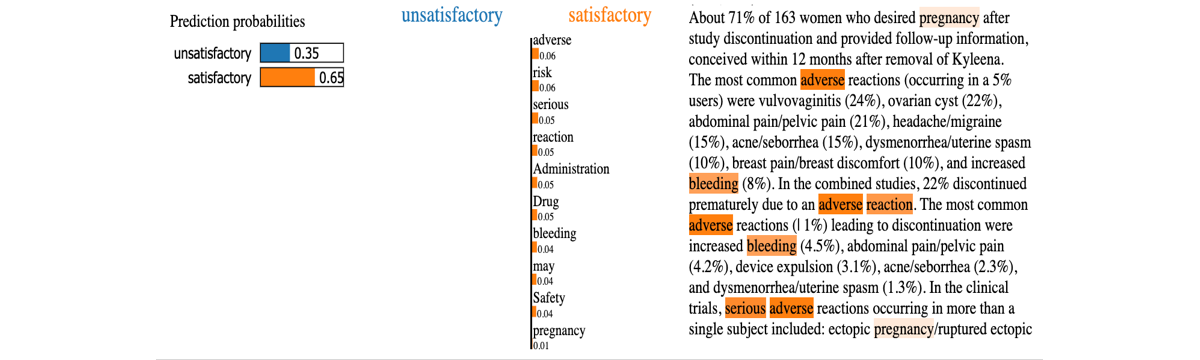
Lime text explainer visualizes word’s contribution to a satisfactory prediction on the harm criterion using random forest algorithm.

**Figure 5:**
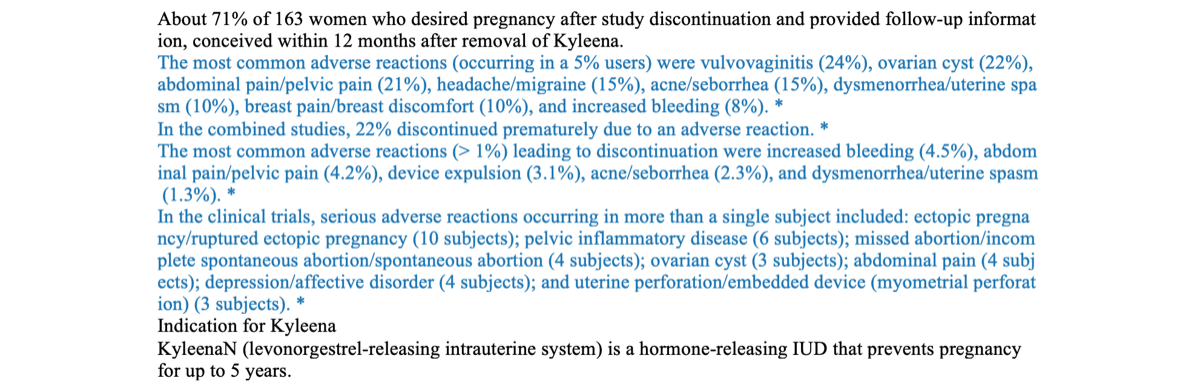
Example of a highlighting scheme for the harm criterion by the hybrid approach.

Figure 4 shows how LIME performed first-level visualization on a sample health news that was rated as satisfactory on the harm criterion. The classifier predicted the sample health news with a positive result of 65% probability. The words marked in orange were picked by LIME and explained as they contributed to the positive classification results of the model. Certain words were also highlighted in blue despite being scarce in number, indicating the likelihood of an unsatisfactory prediction. On the basis of the prediction result, the words “adverse,” “reaction,” “risk,” “adverse,” “serious,” and “administration,” were ranked among the most predictive words in the satisfactory classification result. A snapshot of the final visualized representation is shown in Figure 5, after highlighting sentences containing the keywords selected by LIME and the human expert. The 2-level visual interpretation cases for the cost and conflict criteria can be found in the Multimedia Appendix 2.

#### The Visual Interpretation by the Topology Approach

**Figure 6:**
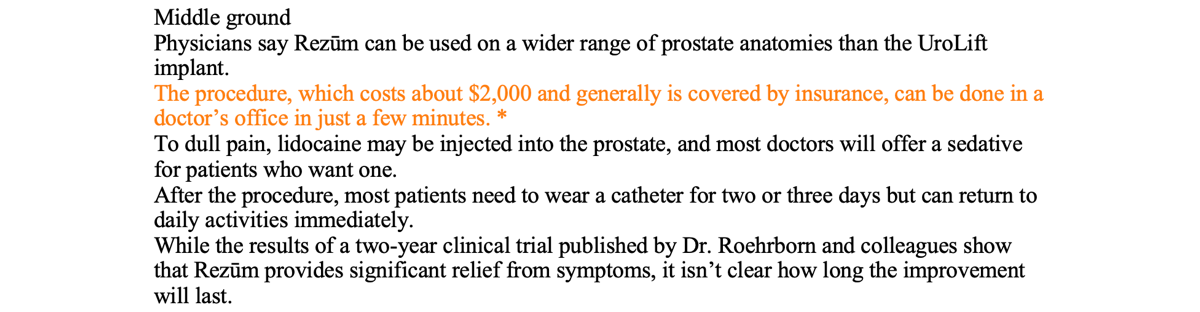
Example of a highlighting scheme for the cost criterion by the typology approach.

The interannotator agreement rates on evidence extraction for the cost, harm, and conflicts criteria were 72.04%, 72.24%, and 77.91%, respectively. The extraction task for each criterion yielded 201 (cost criterion), 318 (harm criterion), and 694 (conflict criterion) sentences in the positive class. We randomly selected the same number of sentences as the negative class to build the classification data sets. Following the same approach applied to the automation of criterion evaluation, which included base classifiers, word feature count selection, and hyperparameters tuning using RandomSearch, the classifiers of the 3 criteria attained an average AUC of 0.8791 (cost criterion), 0.7232 (harm criterion), and 0.8951 (conflict criterion) with 50 repetitions of 10–cross-fold validations. Figure 6 shows the result of applying the classifier to each sentence in the document and highlighting positive sentence instances that supported a cost criterion evaluation.

#### The Overall Performance and Optimization of the 2 Approaches

As the total number of highlighted sentences increased from 1, we calculated the varying rates of accurately highlighted sentences, as shown in Table 3. The numbers with footnotes suggest that the relevant approach could obtain a better result (accuracy >75%) within a certain number of sentences for highlighting.

According to Table 3, the accuracy of both approaches declined as the number of highlighted sentences increased. When both approaches highlighted the same number of sentences, the hybrid approach outperformed the typology approach in most scenarios. Typology, however, performed more accurately when the target was to pick <3 sentences to justify the harm criterion evaluation. When the threshold for highlighting accuracy was set at 75%, the optimal window size for the typology approach to achieve relatively better interpretation results was 2, 4, and 1 for the cost, harm, and conflict criteria, respectively. Comparatively, the hybrid approach still produced comparable outcomes when the window size for each criterion was extended by 2.

**Table 3:** The accuracy of both approaches for interpreting each criterion evaluation; maximum highlighting sentence count.

Number	Criterion and approach							
	Cost			Harm			Conflict	
	Typology (%)	Hybrid (%)	Typology (%)		Hybrid^a^ (%)	Typology (%)		Hybrid (%)
1	80.00^a^	100.00^a^	100.00^a^		90.00	75.00^a^		90.00^a^
2	75.00^a^	92.50^a^	97.50^a^		90.00	72.50		87.50^a^
3	60.78	86.67^a^	86.67^a^		90.00	66.67		81.67^a^
4	66.67	76.25^a^	76.39^a^		85.00	61.11		68.75
5	60.00	67.00	72.94		81.00	55.29		59.00
6	54.55	59.65	66.67		75.83	56.41		50.93

^a^The relevant approach could obtain a better result (accuracy >75%) within a certain number of sentences for highlighting.

## Discussion

### Principal Findings

This study experimented with 2 AI-based approaches to visualize the interpretation of a criteria-based system designed to assist users in systematically evaluating the quality of health news.

The findings of our experiments were 3-fold. First, we found that both the hybrid and typology approaches could achieve the desired visualization result to justify the predicted evaluation result, despite the nature of the 2 approaches being differentiated. With 20 tests for each criterion, the performance of the hybrid approach was slightly better than that of the typology approach. Second, we were able to locate a window size to predetermine the sentences to be highlighted for a better visualization result for each criterion. The hybrid approach showed a higher capacity to reliably choose more sentences when the accuracy criterion was set at 75%. Third, the feasibility of the rule-based strategy to enhance LIME’s interpretation work was supported by our observation during evidence extraction for the typology approach that specific words or phrases such as “adverse effect,” “danger,” “death,” and “side effect” appeared repeatedly in the evaluation of the harm criterion; keywords such as “cost,” “price,” and “insurance” frequently appeared for the cost criterion evaluation; and “spokesman,” “funding,” and “sponsor” were typically used to disclose the conflicts of interests.

#### A Comparison of the 2 Approaches

The hybrid approach demonstrated both good accuracy and efficiency in visualizing the automatic model’s interpretation for evaluating the 3 criteria. Compared with the typology approach, it was advantageous in saving manual effort because it did not require sentence extraction. We also observed that the hybrid approach tended to pick fewer sentences but with higher accuracy when not limiting the maximum number of sentences to be highlighted. By contrast, the typology approach selected more sentences, but only a few were relevant to the criterion.

However, the hybrid approach also had inherent weaknesses. The highlight scheme in the hybrid approach was to locate the sentence in which keywords were present. The drawback of this scheme was that it sometimes failed to discern the semantic differences between a sentence about the risk of the intervention and a sentence that described the benefits of the intervention by relieving or preventing adverse conditions. For example, in one of the test cases, the sentence, “Moreover, the study verified that long-term use of bisphosphonate drugs reduces the risk of typical osteoporosis fractures by 24 percent.” was incorrectly highlighted. The sentence contained keywords, including “risk” and “fractures,” which are relevant to adverse symptoms. However, it introduced how bisphosphonates are expected to benefit patients by decreasing the risk of negative outcomes. The other weakness associated with the hybrid approach was that it failed to distinguish between the intervention and stock prices. Both types of sentences typically shared many keywords that described the values associated with the intervention.

By contrast, the typology approach performed somewhat better at handling expressions with more lexical variations. For example, sentences, “Last fall the Food and Drug Administration issued a ‘safety update’ urging doctors and patients to be on the lookout for the problem.” and “These medications are now linked to a growing number of complications, ranging in seriousness from nutrient deficiencies, joint pain and infections to bone fractures, heart attacks and dementia.” were successfully picked by the typology approach; whereas they were missed by the hybrid approach, as keywords in those sentences were less commonly used to describe side effects. The typology approach distilled relevant information from text documents through sentence extraction by human experts. This information was key to building a knowledge base for the identification of sentences about side effects. We anticipated that the typology approach will be more robust and stable than the hybrid approach when visualizing the interpretation of criteria that are less keyword-reliant. For example, 1 of the 10 criteria, “Does the news compare the new approach with existing alternatives?” examined whether health news included a discussion on alternatives. Sentences that supported a satisfactory evaluation result may have been less likely to be observed with repetitive keywords than with the experimental criteria.

### Limitations

This exploratory study had some limitations. The first limitation was that we only considered the TF-IDF values of words as features for building both the document- and sentence-level classifiers. We acknowledged that the performance of our document-level classification model was lower compared with similar studies that adopted the same data set from HealthNewsReview.org. The performance of our doc-level classification models for the harm, cost, and conflict criteria were 0.71, 0.82, and 0.67, respectively, when measured by *F*_1_ and 0.76, 0.88, and 0.72 when measured by AUC. The performance was better compared with a study by Al-Jefri et al [58] that focused on building health news quality classification models. The precision performance for classifying the harm, cost, and conflict criteria was reported to be 74.61, 77.61, and 70.89, respectively. The study incorporated more features, such as TF-IDF, comparative forms, and named-entity recognition tags and strategically changed the feature selections for different criterion classification tasks. In another study by Afsana et al [59], which also aimed to achieve the same research goal, the performance of their models for the harm, cost, and conflict measures by weighted *F*_1_-score was reported 0.84, 0.899, and 0.835, respectively. However, superior performance was achieved through extensive work on feature engineering with 53,012 features applied. Considering that the key focus of this study was to experiment with 2 interpretation approaches, which both mentioned studies lack, we believed that the current performance of models was effective in serving the purpose of the study. In the future, we will incorporate some work on feature engineering for both document-level classification and especially the typology approach, which is embodied as a sentence-level classifier.

The second limitation is the simple rules of the hybrid approach. The hybrid approach takes advantage of both human knowledge and an autogenerated keyword list generated by the LIME. However, existing rules provided by human experts were keyword-based and did not contain complex rules for handling various expression variants. As part of the future plan, we will implement more complex rules for the hybrid to address the weak spots of the hybrid to enable it to distinguish different types of sentences when they share similar lexicons but different semantics.

A further limitation of the study was the absence of a user study to investigate how the final visual interpretation generated by the 2 interpretation approaches would increase user trust in a black-box model, particularly in the context of evaluating the quality of health news to mitigate misinformation. However, we have an ongoing user study to investigate whether a criteria-based system with visualized interpretation for evaluating health news quality will increase the trust of users compared with the system without interpretation. As of the completion of this study, the user study is still in the recruitment phase.

### Comparison With Prior Work

Our study addressed the public’s need to help evaluate the quality of health news and the typical opaqueness of an AI approach. The significance of this study is illustrated in 2 ways.

First, compared with previous interpretability work in suggested health-related misinformation detection systems, our work on adding the interpretability of a health misinformation system is innovative. To our knowledge, the current state of the art in explainable misinformation detection systems mostly looks to provide explanations for veracity predictions concerning inputs to the system. Our study fills a gap in the literature by explaining a criteria-based system for health misinformation. Moreover, developing an interpretable module on a criteria-based model is advantageous. The criteria-based approach inherently looks for the linguistic characteristics of health news, such as the presence or absence of crucial information, whereas a veracity-based system may face a challenge to be interpreted based on the linguistic features of text alone. In addition, we believe that our study exhibited a greater level of readability of the interpretation than the existing interpretation work on health misinformation, such as Alharbi et al [80] for fake news. The interpretation level achieved in the study by Alharbi et al remained at the word level, with both positive and negative words highlighted and dispersed throughout the articles; whereas our study presented 2 approaches to achieve sentence-level visualized interpretation, which demonstrated higher levels of readability to end users.

Second, this exploratory study demonstrated great potential for the development of a criteria-based system for evaluating the quality of health news as a way to counteract health misinformation. Compared with a veracity-based health misinformation detection system, a criteria-based system demonstrated high generalizability in handling health information on various topics. Most existing veracity-based fake news detectors are built on linguistic cues, leading to a lack of generalizability across topics, languages, and domains [82]. This weakness was also proven in a study by Gerts et al [41], as the team found a huge variation in the classifier performance (*F*_1_-scores between 0.347 and 0.857) on 4 conspiracy topics and more narrowly defined topics could increase performance. In comparison, the idea of a criteria-driven system was to evaluate the quality of health news based on evidence for specified criteria. The evaluation procedure did not require a significant amount of domain knowledge. Thus, this type of system can be adapted to handle a variety of health news stories on various themes, as it did not rely on a data set with a strictly defined topic. In addition, an interpretable, criteria-based system may address the complexity and multidimensional attributes of the health information disorder [83-85]. Automatic tools for evaluating health misinformation have proven promising owing to their high accuracy and fast processing speed. However, existing studies are still predominantly binary classification tasks. This places a great challenge in identifying health misinformation, as the binary label is insufficient to represent the complicated evaluation process of health news in actual practice. This is especially the case with the veracity-based classification. Human-based fact-checking involves extensive knowledge understanding, inference, and source tracking, which remains a challenge, even in deep learning methods. This is because fabricated news is intended to mirror the truth to deceive readers; as a result, without cross-referencing and high-level inference, it might be impossible to determine the authenticity of news stories by text analysis alone [82]. Although it does not provide veracity-level health news validation for users, it has the potential to provide another way of combating health misinformation by improving users’ critical thinking about health news, as the slogan on HealthNewsReview.org indicates.

### Conclusions

In this study, we described an interpretable, criteria-based strategy for evaluating the quality of health news. We explored 2 methods for visualizing the interpretation of the system. To aid in the exploration, an experiment was developed by comparing rule-based and statistical ML approaches. Our results suggested that either approach can successfully automate criterion-based health news quality ratings, with visual evidence supporting model explanation. This study has the potential to increase public trust in computer-assisted reviews of health information. We intend to expand on this study by applying 2 visualization approaches to more criteria and focusing on improving the performance of the classification model.

## Appendix

Multimedia Appendix 1 Performance of different base classifiers for automating 3 criteria evaluation.

Multimedia Appendix 2 The 2-level visual interpretation cases for the cost and the conflict criteria.

## Abbreviations

AI: artificial intelligence
AUC: area under the curve
LIME: local interpretable model-agnostic explanation
ML: machine learning
RF: random forest
TF-IDF: term frequency–inverse document frequency
