# Supplementary material for: Visualizing the Interpretation of a Criteria-Driven System That Automatically Evaluates the Quality of Health News: Exploratory Study of 2 Approaches
Source: JMIR AI. 2022 Dec 20;1(1):e37751. doi: 10.2196/37751 (PMC11041450; doi:10.2196/37751)
Supplement: Multimedia Appendix 1 [file ai_v1i1e37751_app1.docx]

**Multimedia Appendix 1**

**Table 1**. Performance of different base classifiers for automating three criteria evaluation

| **Word Feature  Count** | **Base Classifier** | **Measure** | **the Harm criterion** | **the Cost criterion** | **the Conflict criterion** |  |
| --- | --- | --- | --- | --- | --- | --- |
|  |  |  |  |  |  |  |
| 500 | RF | AUC | 0.6992 | 0.8608 | 0.6886 |  |
|  |  | F1 | 0.7000 | 0.8191 | 0.6421 |  |
|  | SVM | AUC | 0.6920 | 0.8418 | 0.6390 |  |
|  |  | F1 | 0.7049 | 0.8364 | 0.6001 |  |
|  | NB | AUC | 0.6530 | 0.7716 | 0.5852 |  |
|  |  | F1 | 0.6906 | 0.8077 | 0.5620 |  |
|  | LR | AUC | 0.6960 | 0.8272 | 0.6416 |  |
|  |  | F1 | 0.7074 | 0.8221 | 0.6040 |  |
| 1000 | RF | AUC | 0.7202 | **0.8733** | **0.7036** |  |
|  |  | F1 | 0.7054 | **0.8319** | **0.6416** |  |
|  | SVM | AUC | 0.6906 | 0.8372 | 0.6167 |  |
|  |  | F1 | 0.7099 | 0.8280 | 0.5783 |  |
|  | NB | AUC | 0.6613 | 0.7741 | 0.5698 |  |
|  |  | F1 | 0.6881 | 0.8102 | 0.5447 |  |
|  | LR | AUC | 0.6975 | 0.8219 | 0.6283 |  |
|  |  | F1 | 0.7025 | 0.8240 | 0.5813 |  |
| 2000 | RF | AUC | **0.7289** | 0.8671 | 0.7007 |  |
|  |  | F1 | **0.7099** | 0.8220 | 0.6455 |  |
|  | SVM | AUC | 0.6823 | 0.8361 | 0.6254 |  |
|  |  | F1 | 0.7034 | 0.8290 | 0.5828 |  |
|  | NB | AUC | 0.6563 | 0.7748 | 0.5830 |  |
|  |  | F1 | 0.6891 | 0.8107 | 0.5669 |  |
|  | LR | AUC | 0.6965 | 0.8257 | 0.6377 |  |
|  |  | F1 | 0.7025 | 0.8245 | 0.6006 |  |
| 4000 | RF | AUC | 0.7037 | 0.8633 | 0.7001 |  |
|  |  | F1 | 0.7103 | 0.8250 | 0.6505 |  |
|  | SVM | AUC | 0.6776 | 0.8291 | 0.6338 |  |
|  |  | F1 | 0.6941 | 0.8265 | 0.5951 |  |
|  | NB | AUC | 0.6491 | 0.7496 | 0.5917 |  |
|  |  | F1 | 0.6920 | 0.8092 | 0.5625 |  |
|  | LR | AUC | 0.6948 | 0.8239 | 0.6430 |  |
|  |  | F1 | 0.6940 | 0.8191 | 0.6030 |  |
| 8000 | RF | AUC | 0.7140 | 0.8654 | 0.6283 |  |
|  |  | F1 | 0.7054 | 0.8196 | 0.5966 |  |
|  | SVM | AUC | 0.6750 | 0.8115 | 0.6283 |  |
|  |  | F1 | 0.7015 | 0.8230 | 0.5966 |  |
|  | NB | AUC | 0.6293 | 0.7120 | 0.5905 |  |
|  |  | F1 | 0.6915 | 0.7998 | 0.5585 |  |
|  | LR | AUC | 0.6925 | 0.8132 | 0.6354 |  |
|  |  | F1 | 0.6940 | 0.8097 | 0.5951 |  |
