# Supplementary material for: Visualizing the Interpretation of a Criteria-Driven System That Automatically Evaluates the Quality of Health News: Exploratory Study of 2 Approaches
Source: JMIR AI. 2022 Dec 20;1(1):e37751. doi: 10.2196/37751 (PMC11041450; doi:10.2196/37751)
Supplement: Multimedia Appendix 2 [file ai_v1i1e37751_app2.docx]

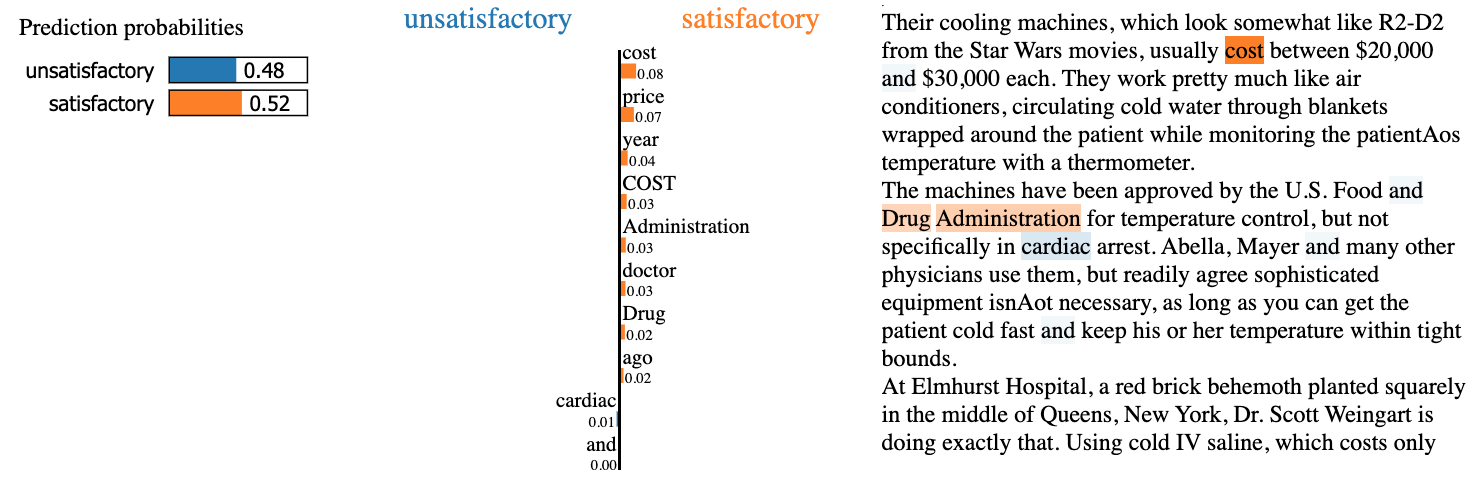


**Figure S1.** LIME text explainer visualizes word’s contribution to a satisfactory prediction on the cost criterion using Random Forest algorithm


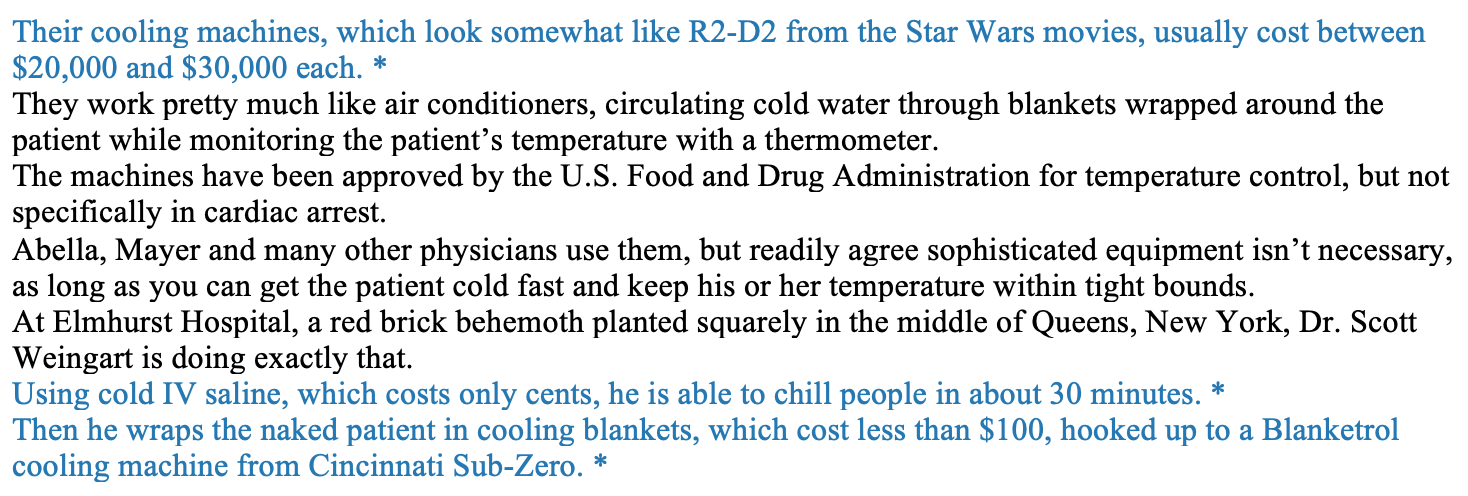


**Figure S2.** Example of a highlighting scheme of the cost criterion by the hybrid approach


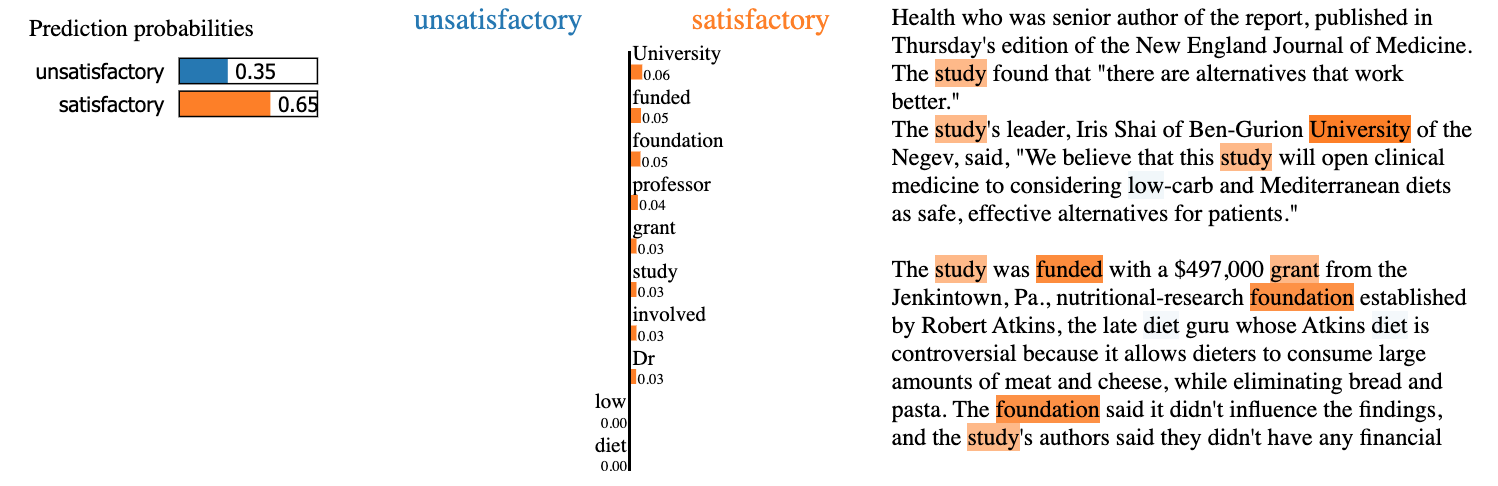


**Figure S3.** LIME text explainer visualizes word’s contribution to a satisfactory prediction on the conflict criterion using Random Forest algorithm


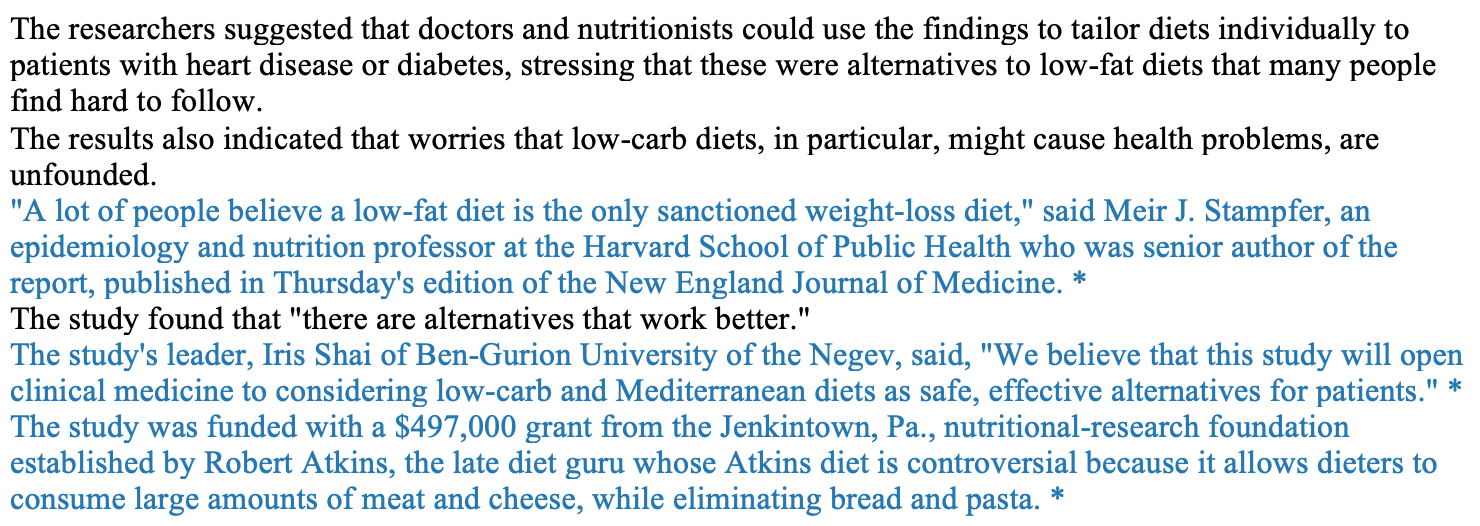


**Figure S4.** Example of a highlighting scheme of the conflict criterion by the hybrid approach
